# Navigating the artificial intelligence-informed patient: a cross-sectional study of physicians’ perceptions and clinical practices

**DOI:** 10.3389/fpubh.2026.1932876

**Published:** 2026-09-23

**Authors:** Malak Almalki, Sama'a AlMubarak

**Affiliations:** 1 Imam Abdulrahman Bin Faisal, Khobar, Saudi Arabia; 2 King Abdulaziz Hospital, Ministry of National Guard Health Affairs, Alhasa, Saudi Arabia

**Keywords:** AI-informed patients, artificial intelligence, health information seeking, perceptions, physicians, practice, Saudi Arabia

## Abstract

**Background:**

Driven by advancements in artificial intelligence (AI) tools, patients are increasingly acquiring medical information independently. While existing literature focus on examining patients’ use of AI tools, understanding physicians’ perspective is essential for patient care. Currently, there is a lack of research examining physicians’ perceptions and clinical practices regarding AI-informed patients, particularly concerning how these dynamics are influenced by physicians’ sociodemographic factors.

**Objective:**

The study aimed to explore physicians’ perceptions and practices towards patients’ use of AI tools for health information seeking. It also assessed how their perceptions and practices differed given physician’s sociodemographic characteristics.

**Methods:**

A cross-sectional study was conducted to assess perceptions and clinical practices related to patients’ use of AI-based tools. Data were collected through an online questionnaire of physicians in Saudi Arabia. Data were analyzed through descriptive and inferential statistics.

**Results:**

A total of 393 physicians participated in the study, in which 66.7% of them encountered AI-informed patients. Overall, 54% were familiar with AI tools, 56.2% believed these tools empower patients, 40.5% reported a positive impact on the patient-physician relationship, and 78.4% identified risks regarding self-diagnosis. The majority of the physicians noted how AI-informed patients shaped their practices in terms of extended consultation time and challenged decision-making processes. Significant differences were found in physicians’ perceptions and practices according to their age, gender, professional position, and healthcare sector.

**Conclusion:**

The findings of the study provide valuable insight to construct policies and guidelines to support the safe and effective utilization of AI-based tools. They also highlight the need for targeted patient awareness initiatives and highlight the need for developing physician training programs to effectively manage AI-informed patients and facilitate adaptation.

## 1 Introduction

Artificial intelligence (AI) is transforming global healthcare by increasing diagnostic accuracy, optimizing treatments, reducing medical errors, and supporting informed decision-making (1, 2). In radiology and pathology, AI systems significantly improve early detection for critical conditions like cancer, neurological disorders, and cardiac diseases, even outperforming human radiologists in detecting abnormalities on mammograms (3–6). Beyond diagnostics, AI drives personalized medicine by tailoring oncology therapies, and it enhances cardiovascular care through real-time wearable monitoring for irregularities like atrial fibrillation (7). These technologies also proved vital during the COVID-19 pandemic for predicting outbreaks and accelerating vaccine development (8). Ultimately, AI has fundamentally changed how physicians diagnose, treat, and manage diseases by serving as a supportive tool for decision-making, treatment planning, and administrative tasks. This drastically reduces operational burdens on physicians, thereby improving quality of care, enhancing interprofessional communication, and mitigating adverse events (9).

To understand how AI tools fit into practice, previous studies have explored physicians’ perspectives regarding the use of AI tools in the medical field. These studies document mixed viewpoints among physicians. Many physicians recognize that AI can streamline administrative burdens—such as optimizing appointment scheduling and providing rapid access to medical information. Conversely, concerns persist regarding privacy, accuracy of diagnosis, ethical considerations, and information bias (10–13). Exploring how these dual perspectives manifest among physicians, Palanica et al. (14) investigated physicians’ perceptions regarding the use of chatbots by patients in the United States. Physicians recognized chatbots’ benefits for scheduling appointment, locating clinics, and providing information. However, they reported that chatbots’ lack of human emotions and inability to account for personal factors hindered its capacity to provide accurate diagnoses or meet patient needs. This dual perspective aligns with Lu and Schulz’s (15) systematic review on internet-informed patients, which found that physicians’ perspectives dictate their communication style. Physicians holding positive views adopted a participative approach that integrates internet information with clinical expertise, while negative views prompted defensive strategies that discouraged online information seeking. However, the utility of these insights is limited, as the included studies were geographically concentrated in Europe and North America and focused primarily on oncologists and general practitioners rather than a diverse range of medical specialties. These documented differences can be theoretically grounded in Street’s Ecological Model of Medical Communication (16), which posits that medical interactions are governed by intersecting cognitive, interpersonal, and contextual factors. Specifically, physicians’ baseline perceptions and communicative orientations actively shape medical encounters and shared decision-making, ultimately dictating whether patient-initiated information is received as a collaborative asset or a relational threat.

Beyond hospital settings, AI has transformed how patients independently seek health information. This practice has long been a primary method for managing new symptoms, medications, or chronic conditions. Rather than navigating traditional websites, patients are shifting towards AI tools like conversational chatbots to evaluate symptoms, monitor their health, seek consultation, and manage ongoing health conditions (17). This shift provides fast and accessible health consultations, enabling individuals to become highly informed and less dependent on physicians for medical knowledge (18, 19). However, heavy reliance on AI raises critical concerns regarding the spread of medical misinformation, the risk of symptom misinterpretation, and the overall impact on patient health outcomes and the traditional patient-physician relationship (20).

Patients use AI-tools to seek health information for several reasons. Chief among these is the increasingly common practice of self-diagnosis. In this practice, patients leverage AI tools to investigate their medical conditions, analyze potential health problems, and determine the next appropriate steps, without consulting their physicians (20). A recent study found that roughly 75% of surveyed individuals in Pakistan utilized AI tools for self-diagnosis (21). Beyond self-diagnosis, patients frequently use AI tools to guide self-medication, a practice that carries significant risks such as delayed clinical consultations and increased likelihood of drug dependence (21, 22). Ultimately, these practices demonstrate that patients’ reliance on AI tools is driven by multidimensional factors, ranging from a need for confidentiality to evade social stigmas of sensitive symptoms to structural barriers such as restricted access to traditional care. Consequently, AI-tools shape patients’ health behaviours by prompting some to book appointments for formal consultations or tests, and others to cancel scheduled visits (18, 23).

Patients’ use of AI tools for health information seeking introduces several benefits. Specifically, it enables easy and effective access to healthcare information, guides informed health decisions, illuminates available treatment options, and fosters patient engagement in managing their health (24, 25). Despite these benefits, AI tools can produce inconsistent or vague results, potentially leading to patient confusion and mistrust. In addition, many AI tools lack personalization as they offer general outputs and overlook personal medical history (20). This limitation is compounded by the inability of AI tools to display human emotions or provide full extent of care needed to meet complex patients’ needs (14). While AI tools offer distinct advantages, they cannot replace trained physicians as they lack human qualities such as empathy, critical thinking, complex decision-making, ethical reasoning, and contextual understanding (26, 27).

Within the context of the global AI widespread, Saudi Arabia provides a compelling case study given its rapid digital health transformation and the strategic integration of AI across its healthcare system under the Saudi Vision 2030. Under the Vision 2030, Saudi Arabia has positioned AI as a cornerstone for public service optimization, particularly through the digital transformation of the healthcare sector to enhance operational efficiency, minimize costs, and improve care quality (28, 29). In 2019, Saudi Arabia established the Saudi Data and Artificial Intelligence Authority (SDAIA) to govern national data and AI initiatives, aiming to accelerate digital transformation, enhance responsible AI adoption across sectors, and position the nation as a global leader in the field (30). Specific to the healthcare sector, Saudi Arabia has launched multiple initiatives for the national digitalization mission, including the adoption of AI solutions and integration of AI into the healthcare sector to improve accessibility and clinical outcomes (31). During the COVID-19 pandemic, the Saudi healthcare system extensively implemented AI applications to mitigate the outbreak which supported outbreak containment through disease monitoring, drug repurposing, rapid data analysis, social media analytics, and early disease detection models (31). In addition, various hospitals in Saudi Arabia utilize AI tools across multiple domains, including medication planning, medical imaging analysis, robotic surgeries, guided radiation therapy, predictive analytics, and oncology treatment planning (32–34). The national commitment to and awareness towards AI are also reflected at the population level. According to the Saudi Center for Opinion Polling, approximately 49% of the Saudi population reported using AI- based tools, with 94% of users perceiving a positive impact of using AI tools on their daily lives (35).

In Saudi Arabia, online health information seeking is a common practice among Saudi patients due to the availability of fast internet and easy accessibility to information from smartphones. Limited healthcare access in rural areas and the stigma surrounding certain medical conditions often drive patients to rely on the internet and AI tools for health information seeking, self-diagnosis, and disease management without seeking clinical consultations (36, 37). Previous studies have documented the active use of AI tools among patients managing chronic conditions such as irritable bowel syndrome and diabetes mellitus. Such studies provide evidence that patients actively modify their behaviors given AI-driven recommendations to enhance health literacy, make informed medical choices, and improve their self-care practices (37, 38).

Physicians in Saudi Arabia hold high levels of awareness towards AI and its applications and benefits in healthcare (39). The majority of studies that have been conducted in Saudi Arabia focus on physicians’ perspectives towards AI adoption in healthcare. Cross-sectional studies were conducted among ophthalmologists (10), otolaryngologists (40) and dermatologists (41), and in various settings including primary healthcare centers (42) and private clinics (43). These studies assessed the knowledge, attitudes, and perceptions of physicians toward AI applications. Findings of these studies indicate that healthcare professionals are highly familiar with AI technologies and generally exhibit positive attitudes toward integrating them into their clinical practice. Most participants believe that AI can minimize clinical errors, decrease practice variations among professionals, and enhance diagnostic speed and accuracy. Furthermore, clinicians recognize AI’s potential to reduce healthcare costs and unnecessary patient visits. Despite these benefits, significant concerns remain regarding the accuracy of AI recommendations, data privacy, and the inherent limitation of these technologies to accommodate individual patient differences, provide clinical sympathy, or account for emotional well-being (10, 40–43).

The extant literature has predominantly explored physicians’ general awareness, acceptance, and perceptions of clinical AI implementation with a narrow focus on specific medical specialties or isolated geographic cities (10, 40, 41, 43). Crucially, no empirical research in Saudi Arabia has yet examined physicians’ perspectives on patients’ use of AI tools for health information seeking and how such behavior shape physicians’ clinical practices. To address this literature gap, this study explores Saudi physicians’ perceptions and clinical practices regarding patients’ utilization of AI tools for health information seeking. Specifically, the study’s objectives were:

1. To assess physicians’ perceptions towards AI-informed patients.
2. To examine how AI-informed patients shape physicians’ practices.
3. To assess how physicians’ perceptions and practices differ given their sociodemographic factors.

The significance of this study lies in providing key insights into the evolving patient-physician dynamic within Saudi Arabia’s transforming digital health landscape. As patients worldwide increasingly rely on AI for health information seeking, understanding the subsequent implications on clinical practice becomes essential, requiring physicians to adapt their skills to effectively evaluate and manage AI-generated data during consultations. Consequently, the findings of the study offer vital takeaways for policymakers tasked with constructing regulatory frameworks and clinical guidelines to ensure the safe and effective utilization of AI within broader healthcare systems. Furthermore, this study informs the development of targeted public awareness campaigns regarding responsible AI use, establishes the groundwork for medical training programs that equip clinicians to manage AI-informed patients, and provides actionable insights for practitioners looking to refine their clinical practices to align with this digital shift. Throughout this manuscript, patients who utilize AI tools to seek health information are referred to as “AI-informed patients.”

## 2 Methods

The study employed a cross-sectional study design. A structured online questionnaire was used to assess physicians’ perceptions and practices regarding patients’ use of AI tools for health information seeking. The study participants included licensed physicians from different specialties and different experience levels who work in Saudi Arabian healthcare facilities. This approach ensured the diversity representation of physicians’ perceptions regarding patients’ use of AI- based tools for seeking health information.

### 2.1 Data collection

Data were collected from January 1 to February 26, 2026, by using a structured online Google Form questionnaire. The questionnaire was distributed via different social media channels such as WhatsApp, Telegram, LinkedIn, and X. A combination of convenience and snowball sampling methods was used. The questionnaire link was initially distributed to physicians, then, physicians were encouraged to share the questionnaire with others. The exact number of physicians who received the invitation could not be determined because the questionnaire was distributed via different applications and could be forwarded to others. Therefore, a response rate could not be calculated. Initially, a total of 430 responses were received, however, only 393 responses met the criteria and were included in the analysis. Before analysis, the dataset was screened for duplicate and incomplete responses. Potential duplicate submissions were identified by examining timestamps of the submission and similarities in response patterns. Around 11 duplicate responses were identified and removed. In addition, approximately 5 respondents were excluded because they did not provide consent to participate. Finally, 21 responses were considered incomplete because respondents did not provide information regarding their medical specialty. Participation was voluntary, and electronic informed consent was obtained from all participants before accessing the online questionnaire. Participants were required to confirm their consent electronically by selecting “I agree to participate” before moving to the questionnaire page. Additionally, the first page contained all the information regarding the study’s purpose, time to complete the questionnaire, voluntary participation, confidentiality, and participants’ right to withdraw at any time.

### 2.2 Study instrument

The study utilized a structured, self-administered, English-language online questionnaire (Appendix in Supplementary material). The questionnaire was developed based on a comprehensive review of the literature and aligned with the study’s objectives. The questionnaire consisted of three sections. The first section covered the sociodemographic characteristics of the physicians, including age, gender, nationality, region of practice, years of experience, position, specialty, type of healthcare sector, and level of healthcare facility. The second section covered physicians’ perceptions regarding patients’ use of AI tools for health information seeking. This section included statements to assess physicians’ familiarity with AI- based tools, their beliefs about AI tools’ reliability, and their perceptions regarding the influence of these tools on the physician–patient relationship. Furthermore, it included a statement to assess physicians’ concerns regarding the potential risks of AI tools. This section used a five-point Likert scale approach ranging from strongly disagree to strongly agree, and participants were asked to indicate their level of agreement with each statement. In addition, this section included one item assessing specific perceived risk types associated with patients’ use of AI tool for self-diagnosis. Using a multiple-response checklist, participants were instructed to select all risk categories that applied. This item was administered to the entire study cohort, regardless of whether they had prior direct clinical encounters with AI-using patients or not.

The last section covered how patients’ use of AI tools shapes physicians’ clinical practices. The first two statements assess how often physicians encountered patients who used AI tools for health information seeking and how often these situations influenced their clinical practice. A five-point frequency scale ranging from never to always was used, and participants were asked to indicate how frequently each situation occurred. The remaining four statements assessed the changes in physicians’ clinical practice in response to patients’ use of AI tools, including influences on clinical decision-making, workload, and consultation time. A five-point Likert scale ranging from strongly disagree to strongly agree was used, and participants were asked to indicate their level of agreement with each statement.

The validity of the questionnaire was assessed through face and content validity. A panel consisting of seven professional and academic experts reviewed and evaluated the questionnaire. They evaluated the clarity, relevance, comprehensiveness, and appropriateness of the questionnaire in relation to the study’s objectives. The panel’s feedback shaped the modifications made including rewording unclear statements, revising language, and refining the wording of questions to ensure clarity and promote participants’ completion of the questionnaire.

A pilot test was conducted among 38 participants to assess the reliability of the questionnaire. The internal consistency was evaluated using Cronbach’s alpha. As a result of distinct constructs of the instrument, Cronbach’s alpha was calculated separately for each subscale. Cronbach’s alpha of the perception subscale demonstrated good internal consistency (*α* = 0.80), whereas Cronbach’s alpha of the practice subscale showed acceptable reliability (*α* = 0.75). These findings indicated that the questionnaire was reliable and suitable for use in the main study. Data from the 38 participants were excluded from the final statistical analysis. Internal consistency was reassessed in the full study sample. Cronbach’s alpha of the perception subscale demonstrated good internal consistency (*α* = 0.86), whereas Cronbach’s alpha of the practice subscale showed acceptable reliability (*α* = 0.73). Using a 95% confidence level and a 5% margin of error, the minimum required sample size was approximately 383 physicians. The final analytical sample comprised 393 physicians, exceeding the minimum required sample size.

### 2.3 Data analysis

The study included two variables of interest–dependent variables. The first variable measured physicians’ perceptions of AI tools used by patients. The composite score for this variable was calculated by summing the responses of the five items within the perceptions domain. The perception scores for the total sample (*N* = 393) ranged from 5 to 22, with a mean score of 14.82 (SD = 3.70). The second variable measured physicians’ practices in response to patients’ use of AI tools. Similarly, the composite score for this variable was calculated by summing the responses of the six items within the practice domain. The practice score for the total sample (*N* = 262) ranged from 10 to 27 (Mean = 21.24, SD = 2.24). The independent variables in this study included the physicians’ sociodemographic characteristics including age, gender, nationality, position, year of experience, specialty, and sector.

The data in this study were analyzed using IBM SPSS Statistics software (Version 26; IBM Corp). Descriptive statistics were conducted to calculate frequencies and percentages. Inferential statistical tests were employed by using an independent samples t-test and one-way analysis of variance (ANOVA) to assess and examine the differences in physicians’ perceptions and practices across sociodemographic groups. Statistical significance was determined at the level of *p* < 0.05 by using a two-tailed alpha test. For statistically significant variables, and to identify specific group differences, *post hoc* pairwise comparisons were performed using Tukey’s HSD test when equal variances were assumed via Levene’s test (*p* ≥ 0.05), or the Games–Howell procedure when equal variances could not be assumed (*p* < 0.05).

The study was approved by the Institutional Review Borad of Imam Abdulrahman Bin Faisal University (IRB-PGS-2025-03-0729).

## 3 Results

### 3.1 Demographic characteristics

A total of 393 physicians participated in this study. Table 1 shows the sociodemographic characteristics of the participants. Almost half of the participants were male (51.9%). The majority of the participants were between 30 and 40 years old (33.6%), while physicians aged 50 years or above accounted for 16.8%. Most of the participants were Saudi (73.5%). Physicians who practiced in the Eastern region represented 40.5% of the participants, while 10.9% practiced in the Southern region. The majority of the participants worked in the government sector (59.3%), and in tertiary healthcare facilities (47.8%). The highest proportion of participants were specialists (42.5%) from internal medicine and related medical specialties (44.8%). Physicians with 10–<15 years of clinical experience represented the greatest proportion of the participants (31.3%). Finally, 66.7% of the physicians reported that within the past 6 months, they interacted with patients using AI tools to seek health information.

**Table 1:** Participants’ sociodemographic characteristics (*N* = 393).

Variable	Variable group	Frequency	%
Age	<30	76	19.3
	30–<40	132	33.6
	40–<50	119	30.3
	≥50	66	16.8
Gender	Female	189	48.1
	Male	204	51.9
Nationality	Saudi	289	73.5
	Non-Saudi	104	26.5
Region of practice	Central	51	13.0
	Eastern	159	40.5
	Northern	50	12.7
	Southern	43	10.9
	Western	90	22.9
Healthcare employment sector	Government	233	59.3
	Private	119	30.3
	Working in both	41	10.4
Healthcare employment facility level	Primary healthcare facility	54	13.7
	Secondary healthcare facility	151	38.4
	Tertiary healthcare facility	188	47.8
Clinical specialty	Internal medicine & medical specialties	176	44.8
	Surgical specialties	105	26.7
	Primary care	43	10.9
	Pediatrics	34	8.7
	Emergency and critical care	16	4.1
	Other*	19	4.8
Professional position	Resident	81	20.6
	Specialist	167	42.5
	Consultant	145	36.9
Years of clinical experience	< 5	76	19.3
	5–<10	120	30.5
	10–<15	123	31.3
	≥15	74	18.9
Have you ever interacted with a patient using AI tools to seek health information in your clinical encounter within the past 6 months?	Yes	262	66.7
	No	131	33.3

*The other category includes: preventive medicine, pain management, and pathology.

### 3.2 Physicians’ perceptions and practices regarding AI-informed patients

Table 2 reports findings on physicians’ perceptions towards AI-informed patients. In this study, more than half of the physicians demonstrated moderate to high familiarity with AI tools, with 54% reporting agreement or strong agreement with the statement. Regarding the physicians’ perceptions towards the reliability of AI-generated health information, 47.1% reported neutral responses, while 32.8% expressed disagreement, and strong agreement was limited to only one participant. Most of the physicians agreed or strongly agreed that AI tools empower patients in making health-related decisions (56.2%). Perceptions of the impact of AI tools on the physician–patient relationship were relatively divided among participants. While approximately 40.5% of participants agreed or strongly agreed that AI tools exert a positive influence, 29.2% disagreed or strongly disagreed, and 30.3% remained neutral.

**Table 2:** Physicians’ perceptions regarding AI tools used by patients for health information seeking (*N* = 393).

Statement, *n* (%)	Strongly disagree	Disagree	Neutral	Agree	Strongly Agree
I am familiar with AI-based health information tools (e.g., symptom checkers, chatbots like ChatGPT).	12 (3.1)	74 (18.8)	95 (24.2)	137 (34.9)	75 (19.1)
I believe that health-related information provided by AI tools is reliable (provides accurate, trustworthy, and consistent information)	21 (5.3)	108 (27.5)	185 (47.1)	78 (19.8)	1 (0.3)
I believe that AI tools empower patients in making health-related decisions.	7 (1.8)	58 (14.8)	107 (27.2)	190 (48.3)	31 (7.9)
I believe that AI tools positively influence the physician-patient relationship (e.g., trust, communication, support, etc.)	15 (3.8)	100 (25.4)	119 (30.3)	143 (36.4)	16 (4.1)
I believe that using AI tools for self-diagnosis poses significant risks to patients	4 (1.0)	7 (1.8)	74 (18.8)	187 (47.6)	121 (30.8)

Physicians reported several risks associated with patients’ use of AI tools for self-diagnosis, as shown in Table 3. This table used a multiple-response format where each participant could select more than one risk; therefore, the total number of responses exceeded the total number of participants (*N* = 545). The column (frequency) shows the number of times each risk was selected by physicians, and the column (percentage) represents the proportion of each response out of the total number of responses (not the total number of participants). Alternatively, the column (percentage of physicians) reflects the proportion of physicians who selected each risk, calculated based on the total sample size (*N* = 393). The most frequently reported risk was incorrect self-diagnosis, accounting for 21.5% of all reported risks and reported by 29.8% of the physicians. The least frequently reported risk was unnecessary medical intervention, accounting for 7.7% of all reported risks and reported by 10.7% of physicians.

**Table 3:** Physicians’ reported risks associated with patients’ use of AI tools for self-diagnosis (*N* = 393).

Risks	Frequency (*N* = 545)	%	Percentage of physicians (*N* = 393)
Incorrect self-diagnosis	117	21.5	29.8
Increased anxiety	90	16.5	22.9
Misinterpretation of health information	84	15.4	21.4
Delayed medical consultation	76	13.9	19.3
Delayed or inappropriate treatment	69	12.7	17.6
Overconfidence in AI recommendations	67	12.3	17.0
Unnecessary medical intervention	42	7.7	10.7

Table 4 reports findings on how AI-informed patients shape physicians’ clinical practices. This section involves results of the 262 physicians who have interacted with AI-informed patients within the last 6 months. More than half of the physicians reported that patients sometimes present with health information obtained from AI tools during clinical encounters (55%), followed by those who reported this occurring rarely (23.7%), while very few indicated it always occurred (1.9%). Among the 262 physicians, 51.9% reported that health information provided by AI-informed patients sometimes influences their clinical encounters, including conducting a consultation or providing a diagnosis. Most of the physicians considered health information obtained from AI tools when presented by patients during their clinical encounters (55.3%). The majority of the physicians reported spending more time in clinical encounters with AI-informed patients (79.4%). Patients’ use of AI tools made the clinical decision-making process more challenging, as reported by 65.7% of the physicians. Finally, the majority of the physicians discuss health information obtained from AI tools with patients during clinical encounter, with 59.2% agreeing and 16.4% strongly agreeing.

**Table 4:** Physicians’ practices given patients’ use of AI tools for health information seeking (*N* = 262).

Statement, *n* (%)	Always	Frequently	Sometimes	Rarely	Never
How often do patients in your clinical practice present health information obtained from AI tools?	5 (1.9)	51 (19.5)	144 (55)	62 (23.7)	0 (0)
How often does information presented by patients from AI tools influence your clinical encounters (e.g., conducting consultation, providing diagnosis)	1 (0.4)	41 (15.6)	136 (51.9)	83 (31.7)	1 (0.4)
Statement, *n* (%)	Strongly disagree	Disagree	Neutral	Agree	Strongly Agree
I consider health information obtained from AI tools when patients present it during clinical encounters	4 (1.5)	18 (6.9)	95 (36.3)	120 (45.8)	25 (9.5)
I spend more time in a clinical setting when patients present health information obtained from AI–based tools.	4 (1.5)	5 (1.9)	45 (17.2)	163 (62.2)	45 (17.2)
I find that patients’ use of AI tools makes my clinical decision-making process more challenging.	3 (1.1)	4 (1.5)	83 (31.7)	133 (50.8)	39 (14.9)
I discuss health information obtained from AI tools when patients present them during a clinical encounter	7 (2.7)	7 (2.7)	50 (19.1)	155 (59.2)	43 (16.4)

### 3.3 Physicians’ perceptions and practices across different sociodemographic characteristics

A structural characteristic of the study’s sample was the near-perfect alignment between young age (<30 years) and early clinical experience (<5 years), which described the same subgroup (*N* = 76). Thus, to address collinearity among demographic variables, a multivariable linear regression was performed with age, years of experience, and professional position entered simultaneously. Multicollinearity was observed between age ( VIF=9.041 ) and years of experience ( VIF=10.439 ). Age emerged as the primary independent predictor of perception scores ( β=−0.384,p=0.001 ), whereas professional position did not reach statistical significance ( β=−.072,p=0.272 ). To eliminate variance inflation and statistical redundancy, years of experience was excluded from demographic comparisons, establishing age as the primary independent variable.

Table 5 reports physicians’ perceptions and clinical practice scores regarding AI-informed patients across different sociodemographic characteristics. *Post hoc* analyses revealed significant sociodemographic variations across physicians’ perceptions and clinical practices (Table 6). Regarding physicians’ perceptions, significant differences were observed according to age, gender, professional position, healthcare sector, and specialty. Specifically, female physicians reported higher mean scores (15.50 ± 3.51) compared to male physicians (14.20 ± 3.77) (*t* = −3.54, *p* < 0.001). In addition, a statistically significant difference was observed across age groups (*F* = 126.01, *p* < 0.001), where younger physicians had higher perception scores. Physicians aged less than 30 years reported the highest mean score (17.76 ± 2.85), while the lowest scores were observed among physicians aged 50 years or above (10.23 ± 2.75). Games–Howell *post hoc* comparisons were performed and showed that physicians aged under 30 years had significantly higher perception scores than those aged 30–<40 years (mean difference = 1.19, 95% CI [0.18, 2.21], *p* = 0.014), 40–<50 years (mean difference = 4.21, 95% CI [3.15, 5.27], *p* < 0.001), and 50 years or above (mean difference = 7.54, 95% CI [6.31, 8.76], *p* < 0.001). (Table 6).

**Table 5:** Physicians’ perceptions and practices scores across different sociodemographic characteristics.

Variable	Category	Perception (*N* = 393)	M	SD	*t*/*F*	*p*	Practice (*N* = 262)	*M*	*SD*	*t*/*F*	*p*
Age	<30	76	17.76	2.85	126.01	< 0.001	46	21.41	2.75	6.14	<0.001
	30–<40	132	16.57	2.45			106	21.51	2.57		
	40–<50	119	13.55	2.66			91	20.27	2.84		
	≥50	66	10.23	2.75			19	19.37	2.14		
Gender	Male	204	14.20	3.77	−3.54	< 0.001	126	20.47	2.68	2.52	0.012
	Female	189	15.50	3.51			136	21.32	2.76		
Nationality	Saudi	289	15.03	3.76	1.91	0.057	204	21.17	2.61	2.89	0.004
	Non-Saudi	104	14.23	3.46			58	20.00	3.04		
Region	Central	51	14.94	3.74	1.32	0.262	38	20.66	3.18	0.26	0.904
	Eastern	159	14.87	3.68			116	20.91	2.92		
	Western	90	14.12	3.86			55	20.91	2.12		
	Northern	50	15.46	3.58			30	20.83	2.53		
	Southern	43	15.21	3.46			23	21.39	2.87		
Sector	Government	233	15.24	3.67	5.19	0.006	157	21.29	2.49	5.51	0.005
	Private	119	14.49	3.52			77	20.05	2.70		
	Both	41	13.39	3.97			28	21.14	3.69		
Facility Level	Primary	54	15.17	4.06	0.27	0.761	31	21.61	2.64	5.37	0.005
	Secondary	151	14.78	3.74			102	20.24	3.21		
	Tertiary	188	14.76	3.57			129	21.27	2.24		
Clinical specialty	Internal Medicine	176	15.16	3.66	3.65	0.003	144	21.21	2.83	1.977	0.082
	Surgical	105	13.90	3.63			57	20.09	2.35		
	Primary Care	43	15.40	4.04			24	21.42	3.16		
	Pediatrics	34	14.97	3.33			24	20.79	2.89		
	Emergency	16	17.00	2.48			5	19.20	1.64		
	Other	19	13.37	3.85			8	21.25	1.28		
Position	Resident	81	17.69	2.81	115.25	< 0.001	46	21.41	2.75	4.782	0.009
	Specialist	167	15.87	2.61			130	21.22	2.40		
	Consultant	145	12.01	3.34			86	20.17	3.11		

**Table 6:** *Post-hoc* pairwise comparisons of physicians’ perceptions and clinical practice scores across sociodemographic characteristics.

Variable	Pairwise comparison (Group I vs. Group J)	Mean difference (I − J)	95% confidence interval	*p*-value
Physicians’ perceptions				
Age	<30 vs. 30–<40	1.19	[0.18, 2.21]	0.014
	<30 vs. 40–<50	4.21	[3.15, 5.27]	< 0.001
	<30 vs. ≥50	7.54	[6.31, 8.76]	< 0.001
	30–<40 vs. 40–<50	3.01	[2.18, 3.85]	< 0.001
	30–<40 vs. ≥50	6.34	[5.30, 7.38]	< 0.001
	40–<50 vs. ≥50	3.33	[2.24, 4.41]	< 0.001
Professional position	Resident vs. Specialist	1.82	[0.94, 2.70]	< 0.001
	Resident vs. Consultant	5.68	[4.69, 6.66]	< 0.001
	Specialist vs. Consultant	3.85	[3.05, 4.66]	< 0.001
Clinical specialty	Emergency and critical care vs. Surgical specialties	3.10	[0.31, 5.90]	0.020
	Emergency and critical care vs. Other	3.63	[0.10, 7.17]	0.040
Sector	Governmental vs. Both sectors	1.85	[0.40, 3.31]	0.008
Physicians’ practices				
Age	<30 vs. ≥50	2.04	[0.16, 3.93]	0.028
	30–<40 vs. 40–<50	1.23	[0.25, 2.22]	0.008
	30–<40 vs. ≥50	2.14	[0.42, 3.86]	0.008
Professional position	Resident vs. consultant	1.24	[0.07, 2.41]	0.035
	Specialist vs. consultant	1.04	[0.15, 1.93]	0.017
Sector	Governmental vs. private	1.23	[0.35, 2.12]	0.003
Facility level	Primary vs. secondary	1.38	[0.07, 2.69]	0.036
	Tertiary vs. secondary	1.04	[0.19, 1.88]	0.012

Table displays only statistically significant pairwise comparisons (*p* < 0.05). Games–Howell *post hoc* test was applied where Levene’s test indicated unequal variances (*p* < 0.05). Tukey’s HSD *post hoc* test was applied where equality of variances was assumed (*p* ≥ 0.05). Non-significant pairwise comparisons were omitted for conciseness.

Statistically significant differences were also observed across physicians’ positions, where residents demonstrated the highest perception scores (17.69 ± 2.81), followed by specialists (15.87 ± 2.61), while consultants had the lowest scores (12.01 ± 3.34), with a statistically significant difference (*F* = 115.25, *p* < 0.001). In relation to the healthcare sector, the results indicated a statistically significant difference (*F* = 5.19, *p* = 0.006). Physicians working in government healthcare facilities reported highest perception scores (15.24 ± 3.67). Furthermore, physicians’ specialty showed a statistically significant difference in perception scores (*F* = 3.65, *p* = 0.003). Physicians working in Emergency and Critical Care reported the highest perception scores (17.00 ± 2.48), whereas lower scores were observed among physicians in Surgical Specialties (13.90 ± 3.63) and other specialties (13.37 ± 3.85). Tukey’s HSD *post-hoc* comparisons showed that physicians in Emergency & Critical Care had significantly higher perception scores than those in Surgical Specialties (mean difference = 3.10, 95% CI [0.31, 5.90], *p* = 0.020) and those in the “Other” specialty category (mean difference = 3.63, 95% CI [0.10, 7.17], *p* = 0.040). No other pairwise comparisons were statistically significant.

The results presented in Table 5 indicate statistically significant differences in physicians’ practice scores according to age, gender, nationality, healthcare sector, facility level, and professional position. In relation to gender, physicians’ practices showed a statistically significant difference (*t* = 2.52, *p* = 0.012), with female physicians reporting higher practice scores (21.32 ± 2.76) than male physicians (20.47 ± 2.68). Likewise, a statistically significant difference was observed according to nationality (*t* = 2.89, *p* = 0.004), where Saudi physicians reported higher practice scores (21.17 ± 2.61) than non-Saudi physicians (20.00 ± 3.04). Physicians’ practices showed a statistically significant difference based on their age group (*F* = 6.14, *p* < 0.001). Physicians aged 30–<40 years reported the highest mean score (21.51 ± 2.57), while the lowest scores were observed among physicians aged ≥50 years (19.37 ± 2.14).

Physicians’ practices varied significantly by their professional position (*F* = 4.78, *p* = 0.009). Residents reported the highest scores (21.41 ± 2.75), while consultants reported the lowest scores (20.17 ± 3.11). Tukey’s HSD *post-hoc* comparisons were performed (Levene’s test, *p* = 0.489). Residents had significantly higher practice scores than consultants (mean difference [MD] = 1.24, 95% CI [0.07, 2.41], *p* = 0.035). Specialists also had significantly higher practice scores than consultants (MD = 1.04, 95% CI [0.15, 1.93], *p* = 0.017). No statistically significant difference was observed between residents and specialists (*p* = 0.905). In addition, physicians’ practices varied significantly across healthcare sectors (*F* = 5.51, *p* = 0.005). Physicians working in government healthcare settings reported the highest scores (21.29 ± 2.49). Finally, a statistically significant difference was observed across facility levels (*F* = 5.37, *p* = 0.005), with physicians working in primary healthcare facilities reporting the highest scores (21.61 ± 2.64).

## 4 Discussion

The study examined how patients’ use of AI tools shaped physicians’ perceptions and clinical practices and investigated differences in physicians’ perceptions and clinical practices according to their sociodemographic characteristics. Overall, the findings indicated that physicians hold favourable perceptions toward AI-informed patients. More than half of the surveyed physicians reported familiarity with AI tools, a finding that aligns with previous studies (10, 41). For example, a recent study found that 82.1% of medical practitioners were familiar with AI tools (44). The documented high levels of familiarity among physicians can be understood in the context of recent national initiatives aimed at accelerating AI adoption and digital transformation across the Saudi healthcare system (39). Furthermore, the study found that many physicians acknowledged the role of AI tools in empowering patients. A recent study supports this perception, indicating that AI tools empower patients to make more informed health decisions by interpreting complex medical information and preparing for clinical consultations (45).

Physicians in this study perceived AI tools as capable of fostering patient empowerment and enhancing the patient-physician relationship, yet simultaneously introducing significant risks. These divergent perspectives mirror a broader debate in recent literature. While some evidence suggests that AI-driven information seeking promotes patient literacy and shared decision-making (44), other scholars caution that widespread access to these tools may propagate misinformation, leading to clinical misunderstandings and a dimension of trust (11). The findings of the study reflect these tensions, with approximately one-third of participants questioning the reliability of AI-generated content. Physicians specifically highlighted risks of incorrect self-diagnosis, increased anxiety, and misinterpretation of health information. These results align with recent studies documenting persistent skepticism regarding diagnostic accuracy, as AI-based systems have been shown to generate imprecise, unspecific, or incomplete medical advice (20, 46, 47). Consequently, physicians remain wary of the potential for these technologies to influence patients toward unsafe health decisions.

Around 66.7% of the physicians reported interacting with AI-informed patients. Recent evidence indicates that patients are increasingly relying on AI applications due to their ease of use and convenient access to medical data. Notably, Alhomoud (48) found that 65% of the surveyed participants utilized AI tools for medication and health-related inquiries. This high level of utilization among the study’s participants can be understood within the broader context of Saudi Arabia as a highly digitalized society. The Saudi population exhibit high levels of internet utilization with internet penetration reaching 99% and mobile usage exceeding 99%. In addition, around 21.5% of the Saudi population actively engages with AI tools, compared to a global average of approximately 16% (49). The Saudi Center for Opinion Polling reported that approximately 49% of the Saudi population uses AI technologies (35). Due to the widespread exposure of AI tools by the population in general, physicians are increasingly interacting with patients who use AI tools.

The study’s findings demonstrate that AI-informed patients actively influence clinical practice by reshaping consultation dynamics, proposing diagnoses, and driving clinical decisions. As physicians actively discuss AI-derived health information with patients, the clinical decision-making process becomes increasingly complex, often requiring extended consultation times. However, this extension may optimize the utility of the clinical encounter, as AI-informed patients can pivot conversational dynamics toward greater productivity, enabling clinicians to bypass basic explanations and focus on specific patient concerns (45). Indeed, these findings suggest that AI-informed patients are fundamentally shifting physicians’ professional roles by expanding and reshaping traditional clinical responsibilities. Instead of acting solely as sources of medical knowledge, physicians increasingly are interpreting and validating AI-generated information, while integrating them into their clinical reasoning. This “new” role contributes to increased physicians’ cognitive burden and consultation complexity, especially given the documented unreliability of AI tools for health information seeking (20, 47). Consequently, clinicians face distinct difficulties when engaging with patients who present with incorrect interpretations. Premature self-diagnosis can introduce interpersonal conflict and impede collaborative decision-making, while the necessary task of correcting and verifying misinformation consumes valuable consultation time that would otherwise be dedicated to treatment planning (20).

A noteworthy finding of the study is how physicians’ perceptions and clinical practices differed across various sociodemographic characteristics. The study found that younger and less experienced physicians exhibited favourable perceptions toward AI tools, which shaped their practices as well. Such findings align with previous studies indicating that younger physicians, as digital natives, view AI tools as a natural extension of clinical practice (50, 51). Older physicians frequently resist digital transformation and demonstrate greater skepticism, leading them to adopt a highly cautious approach toward AI tools (11, 50).

The higher perception and practice scores observed among female physicians regarding AI tools may stem from a more pronounced alignment with collaborative care models. Evidence from extant literature demonstrates that female physicians frequently adopt a partnership-building style that prioritizes patient-centered communication and active patient engagement during consultations (52). Consequently, female physicians may be more receptive to patient-led AI use, viewing it as a valuable asset for shared decision-making rather than a disruption to clinical encounter. Such finding can be interpreted in light of the Medical Communication Model (16). The Model is useful in framing our understanding of gender-based variations on the medical encounter. It explaining how clinicians who naturally utilize collaborative, patient-centered communication styles are structurally more receptive to patient-initiated health information and shared decision-making. Given the study’s findings, the higher receptivity observed among female physicians aligns with collaborative communication models, where patient-initiated AI inquiries are viewed as opportunities for partnership rather than disruptions to clinical authority.

Physicians working in the government sector and primary healthcare facilities demonstrated higher perception scores and significant differences in their clinical practices regarding AI-informed patients. This finding can be understood through the large volume of healthcare services provided by the government sector and primary healthcare facilities, which naturally increases clinicians’ exposure to AI-informed patients and fosters greater operational familiarity with these tools. According to the Saudi Ministry of Health, in 2024 the government sector operated approximately 350 hospitals compared to 166 private hospitals, accounting for roughly 15 million outpatient visits to government facilities alone (53, 54). Similarly, primary healthcare centers serve as the essential first gateway to the healthcare system for a vast majority of the population (55), with previous reports indicating that approximately 85% of all Ministry of Health outpatient encounters occur at the primary care level (56).

## 5 Limitations

While the study examined an underexplored topic in Saudi Arabia, the cross-sectional design limited the study as it does not allow for assessing changes over time or implying causal relationships. The study’s findings have limited generalizability due to possible selection bias and variation in participation across physician groups. For example, participants from the eastern region and from internal medicine and medical specialties were overrepresented in the study’s sample. Also, certain sub-groups, particularly at the clinical specialty level, comprised a small sample size. In addition, the use of a self-administered online questionnaire may introduce response and recall bias. A methodological limitation of this study was the risk of Type I error inflation resulting from conducting 16 independent significance tests across two composite outcomes. Consequently, findings with borderline significance (e.g., perception scores given nationality) should be interpreted with caution as exploratory associations.

## 6 Implications for research, policy and practice

The study’s findings introduce several implications. For future research, it is recommended to use longitudinal and mixed-method approaches to have deeper insights into the impact of patients’ use of AI tools on healthcare practice. For policy, the study highlights the need for developing guidelines and regulatory frameworks to support the safe and appropriate use of AI tools in the health context. Furthermore, the results emphasize the necessity of developing medical training programs to enhance physicians’ AI literacy and prepare them to effectively manage AI-informed patients through targeted education. Finally, for practice, the study provides valuable insights for physicians to adjust, refine, or update their clinical practices to align with the needs of patients who use AI tools.

## 7 Conclusion

The study explored physicians’ perceptions and practices given AI-informed patients. The findings showed that physicians’ perceptions varied across several sociodemographic characteristics. Higher perception scores were associated with physicians” gender, age, position, and clinical specialty. Furthermore, patients’ use of AI-based tools shaped physicians’ clinical practices. Female, Saudi, younger physicians who were in junior positions, and physicians who worked in primary healthcare facilities exhibited differences in their clinical practices in response to patients’ use of AI- based tools. The study introduces several implications for patients, healthcare practitioners and policymakers.

## Data Availability

The datasets presented in this article are not readily available because Survey data that support the findings of this study cannot be shared with third parties because consent for this was not obtained from survey participants. Requests to access the datasets should be directed to shmubarak@iau.edu.sa.
