# Supplementary material for: Navigating the artificial intelligence-informed patient: a cross-sectional study of physicians’ perceptions and clinical practices
Source: Front Public Health. 2026 Sep 23;14:1932876. doi: 10.3389/fpubh.2026.1932876 (PMC13642796; doi:10.3389/fpubh.2026.1932876)
Supplement: Supplementary file 1 [file Table_1.DOCX]

**Appendix**

**Physicians’ Perspectives on Patients’ Use of Artificial Intelligence (AI) Tools for Health Information Seeking: Physician Perceptions and Practices**

Thank you for participating in this survey on patients’ use of artificial intelligence (AI) tools for health information seeking: physician perceptions and practices. This study aims to explore physicians’ perceptions and practices regarding patients’ use of AI tools for health information seeking, examine how this use may influence clinical practice, and assess how these views differ based on sociodemographic factors. Your responses are confidential and will be used solely for research purposes. The survey takes approximately 5–10 minutes to complete. We appreciate your time and valuable input.

**1. I have read the information provided, and I voluntarily agree to participate in this research study.**
○ Yes, I agree
○ No, I do not agree

**Section I: Sociodemographic Information**

The following questions aim to collect general background information about the participants.

**2. Age:**
○ <30

○ 30–<40

○ 40–<50

○ ≥50

**3. Gender:**
○ Male
○ Female

**4. Nationality:**
○ Saudi
○ Non-Saudi

**5. Region of Practice:**
○ Central
○ Western
○ Eastern
○ Northern
○ Southern

**6. Healthcare Facility Employment Sector:**
○ Governmental (Public)
○ Private
○ Working in both

**7. The level of healthcare facilities where you are currently employed:**
○ Primary healthcare facility
○ Secondary healthcare facility
○ Tertiary healthcare facility (e.g., specialty hospital)

**8. Medical Specialty: (Please write your specialty)**

**9. Professional Title / Position:**
○ Resident
○ Specialist
○ Consultant
○ Other: __________________

**10. Years of Clinical Experience:**
○ < 5

○ 5–<10

○ 10–<15

○ ≥15

**Section 2: Physicians’ perceptions regarding AI tools used by patients for health information seeking**

Please indicate the level of your agreement with each of the following statements:

**11. I am familiar with AI-based health information tools (e.g., symptom checkers, chatbots like ChatGPT).**
○ Strongly disagree
○ Disagree
○ Neutral
○ Agree
○ Strongly Agree

**12. I believe that health-related information provided by AI-based tools is reliable (provides accurate, trustworthy, and consistent information).**
○ Strongly disagree
○ Disagree
○ Neutral
○ Agree
○ Strongly agree

**13. I believe that AI-based tools empower patients in making health-related decisions.**
○ Strongly disagree
○ Disagree
○ Neutral
○ Agree
○ Strongly Agree

**14. I believe that AI-based tools positively influence the physician–patient relationship (e.g. trust, communication, support, etc.).**
○ Strongly disagree
○ Disagree
○ Neutral
○ Agree
○ Strongly Agree

**15. I believe that using AI-based tools for self-diagnosis poses significant risks to patients.**
○ Strongly disagree
○ Disagree
○ Neutral
○ Agree
○ Strongly Agree

**16. In your opinion, what is the greatest risk of patients using AI-based tools for self-diagnosis? (You may choose more than one answer.)**
☐ Misinterpretation of health information
☐ Delayed medical consultation
☐ Delayed or inappropriate treatment
☐ Increased anxiety
☐ Incorrect self-diagnosis
☐ Overconfidence in AI recommendations
☐ Unnecessary medical intervention
☐ Other: __________________

**17. If you selected ‘Other’ in the previous question, please specify your response. If you did not select ‘Other’, please write ‘NOT SELECTED’.**

**Interaction With Patients Using AI Tools**

**18. Have you ever interacted with a patient using AI-based tools to seek health information in your clinical encounter within the past 6 months?**
○ Yes
○ No

**Section 3: How AI Tools Shape Physicians’ Practices**

Please indicate the level of your agreement with each of the following statements:

**19. How often do patients in your clinical practice present health information obtained from AI-based tools?**
○ Always
○ Frequently
○ Sometimes
○ Rarely
○ Never

**20. How often does information presented by patients from AI-based tools influence your clinical encounters (e.g., conducting consultation, providing diagnosis....)**
○ Always
○ Frequently
○ Sometimes
○ Rarely
○ Never

**21. I consider health information obtained from AI-based tools when patients present it during clinical encounters.**
○ Strongly disagree
○ Disagree
○ Neutral
○ Agree
○ Strongly Agree

**22. I spend more time in a clinical setting when patients present health information obtained from AI-based tools.**
○ Strongly disagree
○ Disagree
○ Neutral
○ Agree
○ Strongly agree

**23. I find that patients’ use of AI-based tools makes my clinical decision-making process more challenging.**
○ Strongly disagree
○ Disagree
○ Neutral
○ Agree
○ Strongly Agree

**24. I discuss health information obtained from AI-based tools when patients present them during a clinical encounter.**
○ Strongly disagree
○ Disagree
○ Neutral
○ Agree
○ Strongly agree

**Detailed Scoring Instructions**

| **Domain** | **Items Included** | **Scoring Procedure** | **Composite Score Range** |
| --- | --- | --- | --- |
| **Perception Score** | Section 2  Items 11 – 15 | 5-Point Likert Scale (1 = Strongly Disagree, 2 = Disagree, 3 = Neutral, 4 = Agree, 5 = Strongly Agree)  2. Sum recoded scores across items P1–P5. | **Possible range from 5 to 25**  Actual sample range from 5 to 22, (Mean= 14.82,SD = 3.70) |
| **Practice Score** | Section 3  Items 19 - 24 | Response format: 5-Point Likert Scale (1 = Strongly Disagree/ Never, 2 = Disagree/ Rarely, 3 = Neutral/ Sometimes, 4 = Agree/ Frequently, 5 = Strongly Agree/ Always) | **Possible range from 6 to 30**  Actual sample range from 10 to 27 (Mean = 21.24, SD = 2.24). |
